# IVF: The women who helped make it happen

**DOI:** 10.1016/j.rbms.2018.11.002

**Published:** 2018-12-14

**Authors:** Martin H. Johnson

**Affiliations:** Anatomy School and Centre for Trophoblast Research, Department of Physiology, Development and Neuroscience, Downing Street, Cambridge CB2 3DY, UK

**Keywords:** history, women, Jean Purdy, Muriel Harris, Lillian Lincoln Howell, Ruth Fowler

## Abstract

In this paper I pay tribute to four named women and 280 unidentified women patients for their essential roles in supporting Bob Edwards and Patrick Steptoe during the pioneering early days of IVF. The four named women are Jean Purdy, Muriel Harris, Lillian Lincoln Howell and Ruth Fowler.

## Introduction

Much has been written about the achievement of Bob Edwards that led to the award of the Nobel Prize for Physiology and Medicine in 2010. And Edwards was undoubtedly a remarkable man of vision, energy and determination in the face of considerable opposition (Johnson, 2011, Johnson, 2018). However, he was well supported in his work, not just by clinician Patrick Steptoe, another remarkably skilled and determined individual (Edwards, 1996), but also by a host of less-known and under-acknowledged women, about which this article is concerned. It is perhaps fitting in the year that marks the 40th anniversary of the birth of Louise Brown as well as 100 years since women in the UK gained partial voting rights, that the key roles of these women should be emphasized and acknowledged.

### Jean Purdy – laboratory assistant (25 April 1945–16 March 1985)

**Fig. 1:**
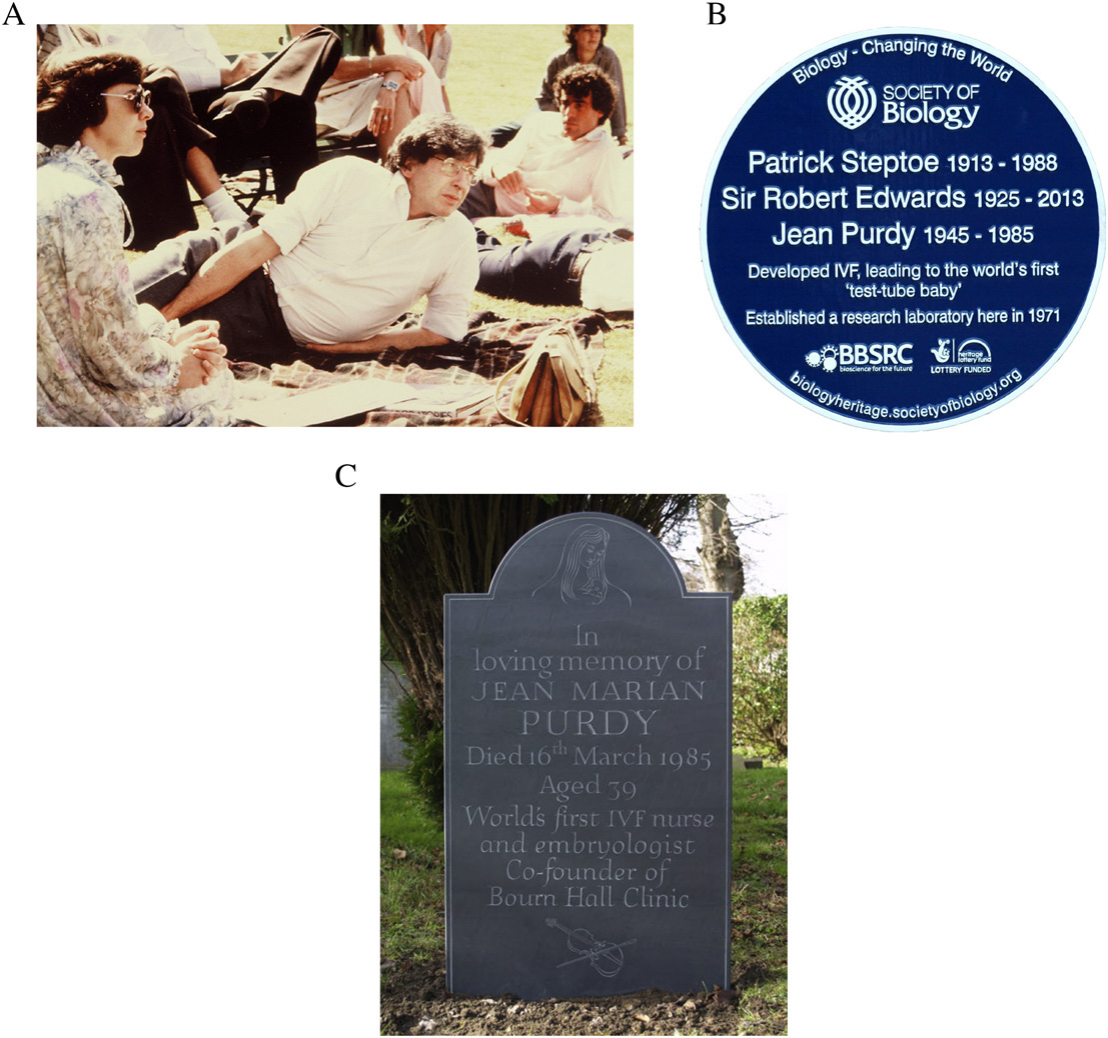
(A) Jean Purdy with Edwards and Steptoe at the first Bourn Hall meeting 1981 (courtesy Bourn Hall Clinic). (B) Blue plaque unveiled on 19 March 2015 at Dr. Kershaw's Hospice (formerly Hospital; courtesy of John Fallows, who has copyright). (C) Tomb stone of Jean Purdy in Granchester churchyard, which replaced the original in 2018.

Perhaps the best known of these women is Jean Purdy, who joined Edwards in 1968 and worked closely with him until her early death from malignant melanoma in 1985 (Fig. 1A). Purdy was registered as a nurse on the 5 December 1966 after three years' training at Addenbrooke's Hospital in Cambridge, and her essential role in the work leading up to the birth of Louise Brown in 1978 was recently identified as recording and organizing most of the data systematically, almost certainly spending longer working in Oldham than did Edwards, and whilst there, taking primary responsibility for organizing laboratory supplies, including media preparation and testing and being involved in patient care (Johnson and Elder, 2015a). Purdy also was identified as being a major source of support to Edwards, in particular when his spirits were very low in the spring and early summer of 1974, when he considered giving up on IVF work, discouraged by the lack of progress and funding, and his weariness with the continued criticism and spending so much time away from his family travelling to and from Oldham (Edwards and Steptoe, 1980, pp. 97 and 122–125). Indeed, in an interview with Purdy's childhood friend, Rosemary Carter, she said that Edwards had told her that Purdy was really vital in keeping the work on IVF going. Thus, when he was becoming very negative about whether the work was going to be able to continue, he had offered Purdy a choice of continuing with IVF or focusing instead on another project on which he was working, namely ‘revitalising the blood’. She claimed that Purdy replied very firmly that she really wanted to continue with IVF, and they went with that (Johnson and Elder, 2015a). Further evidence of Purdy's essential role in the venture comes from the observation that all work ceased between July 1974 and February 1975, the time of Purdy's mother's terminal illness (Johnson and Elder, 2015a). Indeed, Edwards (Edwards and Steptoe, 1980, p.122) described Purdy at this time as being ‘particularly good with the patients’, and that her ‘cooperation had become crucial. It was no longer just Patrick and me. We had become a threesome’. This reevaluation of Purdy's role has led to her being acknowledged publically with Edwards and Steptoe as a pioneer in the development of IVF (Fig. 1B,C; Steptoe, 2015, Gosden, 2017). Purdy was also largely instrumental in locating and organizing the adaptation of Bourn Hall as the locus of the continuing work following Louise Brown's birth, after the National Health Service declined to support the work.

## Muriel Harris – operating theatre superintendent (4 June 1923–14 December 2007)

**Fig. 2:**
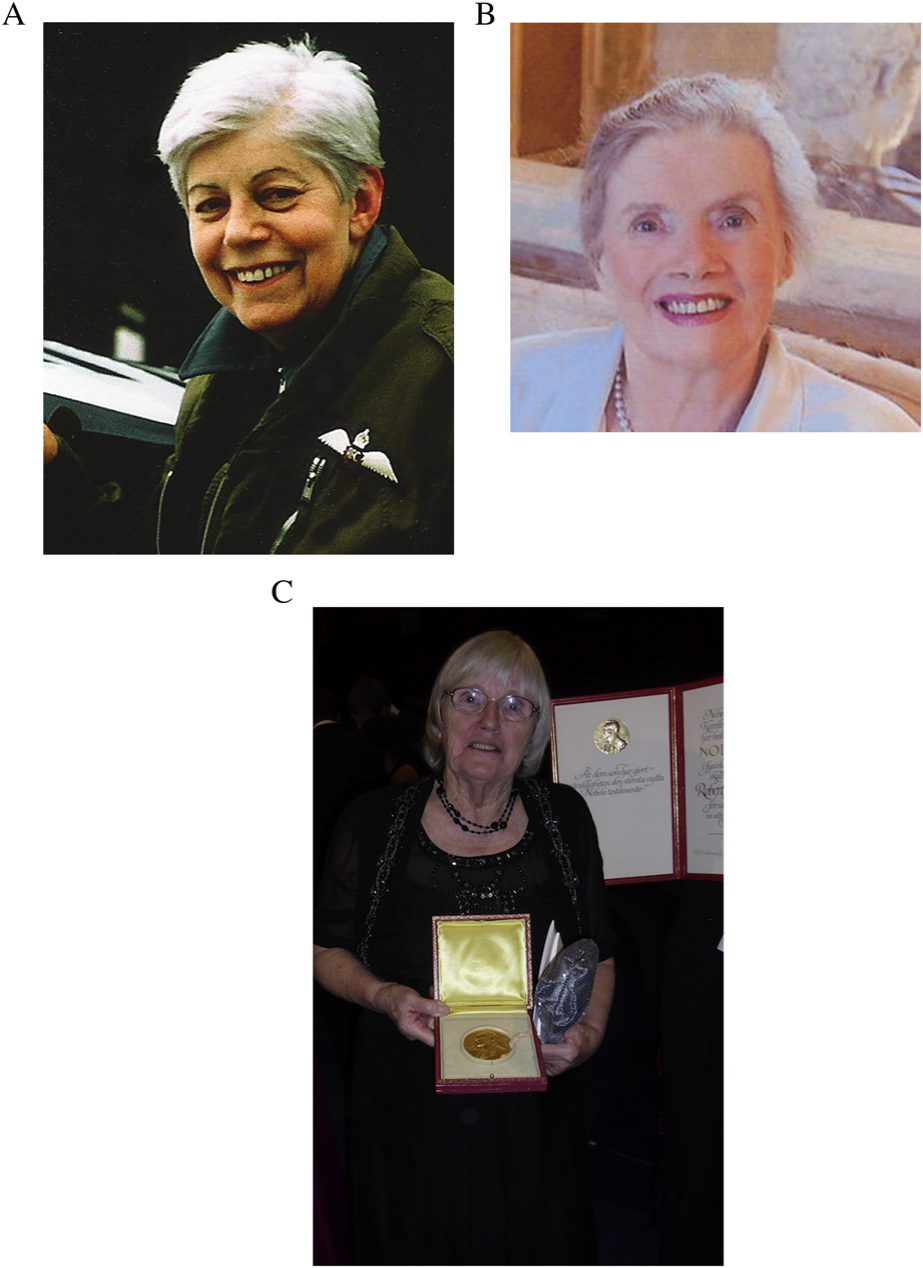
(A) Muriel Harris (courtesy of John Fallows, who has copyright); (B) Lillian Lincoln Howell (from her obituary at http://hosting-19478.tributes.com/obituary/show/Lillian-Lincoln-Howell-101686506); (C) Ruth Fowler-Edwards holding the Nobel Prize, having just received it on behalf of Edwards in 2010 (courtesy of the Edwards family).

In addition to Purdy, the role of the staff at Oldham General Hospital was crucial in the success of the birth of the first ‘test tube babies’. As detailed in Johnson and Elder (2015b), these staff worked unpaid at very antisocial hours to advance the cause of IVF, in particular the operating theatre superintendent, Muriel Harris (Fig. 2A), who was born in Swinton on 4 June 1923, educated at Pendleton High School for Girls, and then at Manchester University, where she gained a BSc. She then studied from 1942 at the London Hospital during the London ‘blitz’ of World War II to become a State Registered Nurse. In 1968 she joined Steptoe and in 1971 equipped for IVF work, on a shoe-string budget of £250, the operating theatre at his newly established IVF clinic at Dr. Kershaw's Hospital in Oldham. She also raised and organized a team of ‘volunteer’ unpaid nursing staff, amongst whom the ‘lynchpins’ were Noni Fallows and Sandra Corbett, to assist Steptoe and Edwards in their attempts to try to recover eggs for fertilization. Unfortunately, Harris was not present at the birth of Louise Brown. She was on holiday in Cornwall when Steptoe scheduled a caesarian delivery on Saturday 26 July. She was driving back to attend when she heard on the radio that Steptoe had moved the birth earlier to 25 July to circumvent the intense media interest. Before Louise Brown was born there were many disappointments and at times the staff became very disheartened, but Muriel, as well as providing nursing support, provided a great deal of moral support. She had a positive outlook, an attitude which helped everyone.

The IVF programme in Oldham closed shortly after the birth of Louise when Steptoe retired from the NHS. The team then went on to open Bourn Hall, the world's first IVF clinic, in 1980. Muriel became the matron of Bourn Hall and she not only interviewed for nurses, housekeeping and cleaning staff but also equipped the operating theatre and the wards in Portakabin units. The place had been empty for some time so Muriel, with a number of her loyal colleagues, got together with the domestic staff to give the place a thorough clean!

After retiring from the Clinic, she gained her Private Pilot License, and flew light planes until she was 80 years of age. She died on 14 December 2007 aged 84 and was cremated in Cambridge followed by a service at Bourn Church (where Steptoe is buried) at which Edwards gave the eulogy.

John Webster, who worked with Muriel at both Oldham and Bourn described her as a “‘one off’ – a great nurse who was blessed with superb organisational skills, and her input toward the IVF programme both in Oldham and Bourn Hall was immense. Without Muriel's support, who knows what may have happened.” https://blog.sciencemuseum.org.uk/muriel-harris-nursing-ivf-to-success/.

## Lillian Lincoln Howell – philanthropist (1921–31 August 2014)

Also important in the quest for successful IVF was Lillian Lincoln Howell, a wealthy Californian heiress, who provided considerable financial support for the work from 1968 onwards (a minimum of $95,000 in total between 1969 and 1978, which equates to £39,700–£49,500 at current rates; Johnson and Elder, 2015b). These funds became essential when the UK Medical Research Council (MRC) actively failed to support the work (from 1971 to 1983; Johnson et al., 2010), and during the period when the Ford Foundation appeared to cease supporting the replacement of embryos in the uterus (1973–1978; Johnson and Elder, 2015b). Howell is described anonymously in Edwards (1986, p.12), as ‘herself having suffered problems similar to those of the patients now being treated’, most probably, given the emphasis of Edwards’ work at the time of her first donations in the late 1960s (Johnson, 2011), being problems of a genetic nature rather than of infertility. Howell was born in 1921 in Cleveland Ohio, the eldest of three children. Her father, John Cromwell Lincoln was a self-made industrialist, born of an itinerant pastor originally from England (Love, 2011). His wealth allowed him to be a generous benefactor, his philanthropic activities including the establishment of charitable trust, the Lincoln Foundation. Lillian Lincoln, who inherited substantially from her parents on their deaths, attended Pomona College, California, between 1939 and 1943, where she studied science and philosophy. After graduating, she taught preschool in California, worked as a recreational director in a disabled children's home in Phoenix, and married her first husband, Carl Howell, with whom she had her only son, Lincoln C Howell, in South Pasadena. The couple separated in 1957, and she later married Deane De Vere Banta, a media executive with whom she bought the license for KTSF, a television channel serving the San Francisco Bay area. After their divorce, she kept the station as part of the settlement so that she could continue the philanthropic tradition of her father, KTSF being one of the nation's first multi-ethnic stations, broadcasting news and entertainment in 12 languages to an audience of about 1.4 million. The business had a strong charitable aspect to it, and did not return a profit until 1986, providing airtime for fund-raising and providing funds itself via the Lillian Lincoln foundation (Hua, 2006; https://www.lillianlincolnfoundation.org/our-mission/).

In addition to supporting Edwards' work, she also supported the research by Dr. Emmet Lamb, lately an emeritus member of Stanford's Department of Obstetrics and Gynecology (personal communication from Lincoln C Howell, March 2011), as well as actively supporting the state legislation bills (SB322, SB771 and SB778) that set up a specific framework for enhancing stem-cell research in the state of California. She said in 2003 “The benefits of stem-cell research have recently been articulated in a variety of rational forums and symposiums. Distinguished American medical researchers … suggest that, simply put, generating new sources of human stem cells will assist us in determining if the bad cells causing Alzheimer's disease, Parkinson's disease, type-1 diabetes, spinal cord injuries and breast cancer can be replaced by healthy ones. How can we not pursue the possible cures for illnesses that have so troubled humankind? If we had followed the argument of some, we would not have sought the cures for tuberculosis, smallpox and cancer plaguing our population for generations” (Howell, 2003). Howell shunned any publicity for her support for Edwards, and both Edwards and his wife Ruth were adamant that this wish should be respected in her lifetime. However, her death on 31 August 2014 (Obituaries, 2014; Fig. 2B), legitimately allowed her identity to be revealed (Johnson and Elder, 2015b).

## **Ruth Fowler – scientist and wife** (14th December 1930 - 3rd October 2013)

The roles of the three women mentioned above have at least been acknowledged previously, unlike those of the women that follow. Ruth Fowler, Edwards' wife and long-time collaborator, was a woman of great intellectual and moral strength, unsurprisingly given her background. She was the grand-daughter of Earnest Rutherford, who himself won the Nobel Prize for chemistry in 1908, ‘for his investigations into the disintegration of the elements, and the chemistry of radioactive substances’ (Eve and Chadwick, 1938). Her father was Sir Ralph Fowler FRS (1889–1944; Milne, 1945), who was Plummer Professor of Mathematical Physics in Cambridge from 1932 to 1944. He was described as being an exceptionally talented mathematical physicist, a fine sportsman and ‘an inspirational teacher and leader of men’ (Milne, 1945). Back in Cambridge in 1919 after World War I, he was stimulated to work with Rutherford, who had recently arrived there to take the chair of Experimental Physics. In the course of doing so, Ralph Fowler met Rutherford's only daughter, Eileen, whom he married in 1921. They had four children, of whom Ruth was the youngest, born in December 1930. Tragically her mother died shortly afterwards and her father moved the family to Cromwell House in Trumpington, Cambridge, which was shared with the Cook family, the children of both families being raised together by Mrs. Phyllida Cook who was the only ‘mother’ Fowler knew (Milne, 1945). Her father, although himself unwell, was to undertake gruelling high security war work at the Ordnance Board and later at the Admiralty during World War II. His health deteriorated and he died at the relatively young age of 55 when Ruth Fowler was 13, having had an unhappy childhood.

Fowler married Edwards in 1954, having first met him, according to Edwards, in a statistics class at the University of Edinburgh in 1952 when she was working on her PhD on genetics, after completing a biology degree there. Edwards was also working on his PhD and the two joined forces to overcome a problem Edwards had encountered. Together, they devised ways of increasing the number of synchronized eggs recoverable from adult female mice through a series of five papers (1957–1961), the first published in 1957 (Fowler and Edwards, 1957), on the control of ovulation induced by use of exogenous hormones. In doing so, they overturned the conventional wisdom that superovulation of adults was not possible. Then, following a year of post-doctoral work in California, the two of them returned to the UK, where Edwards had taken a post at the MRC institute at Mill Hill working on the development of immunological approaches to fertility control. As Fowler was now pregnant with their first child, she stopped her own research work and focused on family. Their five daughters were born between 1959 and 1964: Caroline, Jenny, Sarah, and twins Anna and Meg. During this time she supported the growing family and Edwards during the long periods he spent away from home in the laboratory, travelling to and from Oldham and attending conferences overseas. Fowler re-entered the laboratory to work during the early 1970s on matters reflecting her husband's scientific passion. Her scientific contributions shifted accordingly, publishing papers on the growth of human embryos in the laboratory, the genetics of early human development and on the progesterone and protein composition of the uterine fluids of the rabbit – of importance in understanding the environment experienced by the preimplantation embryo. During this period Fowler became interested in follicular growth and follicular steroidogenesis, and published a series of papers studying mouse, rat and human ovaries. In particular during 1978, the year of the birth of Louise Brown, she published influential papers in collaboration with Edwards on the dynamics and endocrinology of follicular development (Fowler et al., 1977, Fowler et al., 1978a, Fowler et al., 1978b, Fowler et al., 1978c), an interest that continued to her last publication in 1989. In the words of Fishel (2014), who wrote in her obituary: “She was a remarkable woman who had that rare capacity to juggle three, indeed four, of the most difficult and all too often competing roles in our complex lives: successful mother, wife and scientist whose highly significant work spanned more than three decades. And for that “elite fourth” role, provided sustenance at both the intellectual and family level to a great, Nobel Laureate husband!|” It was fitting therefore that when Edwards was too ill to receive the Nobel Prize in person, these words were spoken, “In the absence of this year's Nobel Laureate in Physiology or Medicine, I ask Professor Edwards' wife and long-term scientific companion, Dr Ruth Fowler Edwards, to come forward and receive his Prize from the hands of His Majesty the King” (Fig. 2C).

## The 282 women patients

Finally, the last, but by no means least, women whose roles were so important in the successful achievement of IVF are the patients who underwent the early attempts at treatment between 1969 and 1978, of whom only two are known: namely Lesley Brown, the mother of Louise, and Grace MacDonald, the mother of Alistair (Elder and Johnson, 2015a; supplementary materials). The remaining 280 anonymous women who underwent hormonal stimulation and laparoscopic egg recovery under general anesthesia, but did not become pregnant – at least then – deserve special mention (Elder and Johnson, 2015a), especially the few (7%) women who put themselves forward for four or more attempts. Among these women is included patient 38 who, after experiencing an ectopic pregnancy in 1975 on her third attempt at IVF, continued unsuccessfully for a further seven attempts (Elder and Johnson, 2015a, Elder and Johnson, 2015b). Many of these women do not want to be known, because it is now easy to forget that until recently infertility was stigmatized and was not discussed openly, further emphasizing their courage in undergoing the then novel treatment. All the evidence that we have supports the view that these women were treated ethically (Johnson and Elder, 2015c), and they deserve our deepest thanks and respect, for without their willing involvement, IVF would never have been possible. As Andrew Steptoe (2015), Patrick's son, recalled “Most of all, I think about the infertile women themselves. They came year after year to Kershaw's to undergo the processes of egg removal and embryo transfer. Although they will have harboured hope that they would be lucky, most must have known that there was next to no chance of success for them personally. But they willingly gave themselves so that others would benefit in the future. I vividly remember coming with my father to Kershaw's on my visits from the south of England, driving over with him from my parent's home in Rochdale, often late in the evening to observe oocyte removal or embryo transfer. What struck me was the patient cheerfulness of the women who underwent these procedures, their utter confidence in Patrick and the scientific team, and the kindness with which they were treated by the nurses and other staff here.”

## Discussion

In this article I have tried to indicate those relatively under-acknowledged women without whom Edwards would not have succeeded in achieving IVF. Despite his imagination and commitment when confronted with extraordinary technical problems and also severe professional and social criticism (Johnson, 2018), the support of these women was essential, as he was the first to acknowledge. Thus, the paper restores these women to the historical record. It also challenges common ways of thinking about progress in science and in the clinic as being driven mainly by individuals with the right credentials, which include gender and class, of which Edwards was helped by his gender, but hindered by his class (Johnson et al., 2010). In particular, it acknowledges the epistemic role of patients. The paper hopefully raises general issues about how credit is attributed and how the archive of the history of science is curated. In this context, the Edwards archive is well placed to make a difference on these issues for scholarship and how we think about the history of (British) science more generally.
